# Comorbidity status of deceased COVID-19 in-patients in Italy

**DOI:** 10.1007/s40520-021-01914-y

**Published:** 2021-06-24

**Authors:** Davide Liborio Vetrano, Clare Tazzeo, Luigi Palmieri, Alessandra Marengoni, Alberto Zucchelli, Cinzia Lo Noce, Graziano Onder, Italian National Institute of Health Covid-Mortality Group, Luigi Palmieri, Xanthi Andrianou, Pierfrancesco Barbariol, Antonino Bella, Stefania Bellino, Eva Benelli, Luigi Bertinato, Stefano Boros, Gianfranco Brambilla, Giovanni Calcagnini, Marco Canevelli, Maria Rita Castrucci, Federica Censi, Alessandra Ciervo, Elisa Colaizzo, Fortunato D’Ancona, Martina Del Manso, Corrado Di Benedetto, Chiara Donfrancesco, Massimo Fabiani, Francesco Facchiano, Antonietta Filia, Marco Floridia, Fabio Galati, Marina Giuliano, Tiziana Grisetti, Yllka Kodra, Martin Langer, Ilaria Lega, Cinzia Lo Noce, Pietro Maiozzi, Fiorella Malchiodi Albedi, Valerio Manno, Margherita Martini, Alberto Mateo Urdiales, Eugenio Mattei, Claudia Meduri, Paola Meli, Giada Minelli, Manuela Nebuloni, Lorenza Nisticò, Marino Nonis, Graziano Onder, Lucia Palmisano, Nicola Petrosillo, Patrizio Pezzotti, Flavia Pricci, Ornella Punzo, Vincenzo Puro, Valeria Raparelli, Giovanni Rezza, Flavia Riccardo, Maria Cristina Rota, Paolo Salerno, Debora Serra, Andrea Siddu, Paola Stefanelli, Manuela Tamburo de Bella, Dorina Tiple, Brigid Unim, Luana Vaianella, Nicola Vanacore, Monica Vichi, Emanuele Rocco Villani, Amerigo Zona, Silvio Brusaferro

**Affiliations:** 1 Aging Research Center, Department of Neurobiology, Care Sciences and Society, Karolinska Institutet, Tomatebodavägen 18A, 10th floor17165 Solna, Stockholm, Sweden; 2 Centro Medicina dell’Invecchiamento, Fondazione Policlinico “A. Gemelli” IRCCS and Università Cattolica del Sacro Cuore, Roma, Italy; 3 Department of Cardiovascular, Endocrine-Metabolic Diseases and Aging, Istituto Superiore di Sanità, Roma, Italy; 4 Department of Clinical and Experimental Sciences, University of Brescia, Brescia, Italy; 5 Department of Information Engineering, University of Brescia, Brescia, Italy

**Keywords:** COVID-19, Multimorbidity, Chronic disease, Comorbidity, Mortality

## Abstract

**Background:**

Most COVID-19-related deaths have occurred in older persons with comorbidities. Specific patterns of comorbidities related to COVID-19 deaths have not been investigated.

**Methods:**

A random sample of 6085 individuals in Italy who died in-hospital with confirmed COVID-19 between February and December 2020 were included. Observed to expected (O/E) ratios of disease pairs were computed and logistic regression models were used to determine the association between disease pairs with O/E values ≥ 1.5.

**Results:**

Six pairs of diseases exhibited O/E values ≥ 1.5 and statistically significant higher odds of co-occurrence in the crude and adjusted analyses: (1) ischemic heart disease and atrial fibrillation, (2) atrial fibrillation and heart failure, (3) atrial fibrillation and stroke, (4) heart failure and COPD, (5) stroke and dementia, and (6) type 2 diabetes and obesity.

**Conclusion:**

In those deceased in-hospital due to COVID-19 in Italy, disease combinations defined by multiple cardio-respiratory, metabolic, and neuropsychiatric diseases occur more frequently than expected. This finding indicates a need to investigate the possible role of these clinical profiles in the chain of events that lead to death in individuals who have contracted SARS-CoV-2.

**Supplementary Information:**

The online version contains supplementary material available at 10.1007/s40520-021-01914-y.

## Background

Within the first year since its inception, the coronavirus disease 2019 (COVID-19) pandemic has been responsible for over 2 million premature deaths, particularly among older individuals [1–3]. Italy is among the countries with the highest excess mortality [4, 5]; as of December 16th, 2020, 63,573 persons had died of COVID-19 with a mean age of 80 years [5]. Most persons who have died of COVID-19 were affected by multimorbidity, the co-occurrence of two or more chronic conditions in the same individual [6–8]. A previous report regarding the pre-infection health status of deceased persons in Italy showed that approximately 84% of these individuals had multimorbidity, and that ischemic heart disease and atrial fibrillation were the most common chronic diseases [5]. Several other studies have confirmed that chronic diseases are associated with adverse outcomes in COVID-19 patients [9]. It is also well known that chronic diseases tend to cluster together in the same individual exceeding a level expected by chance alone for several reasons, including shared risk factors and similar pathophysiology [10]. We hypothesized that persons who died from COVID-19 and were affected by multimorbidity had specific disease combinations which co-occurred more frequently than predictable by chance. We aimed to test this hypothesis in a sample of in-patients in Italy with a confirmed diagnosis and related death due to COVID-19.

## Methods

### Study population and data collection

The study population consisted of a nationally representative random sample of 6085 individuals deceased in-hospital, in Italy, with confirmed COVID-19. COVID-19-related deaths were defined as those occurring in patients who tested positive for SARS-CoV-2 through reverse transcription polymerase chain reaction, independent of pre-existing diseases that may have caused or contributed to death [8, 11]. Data collection was carried out between February 2020 and December 2020. As part of the Italian National Institute of Health (Istituto Superiore di Sanità; ISS) COVID-19 surveillance system, the following information was extracted from the participants’ hospital charts: demographic characteristics, COVID-19 symptoms and complications, received treatments, SARS-CoV-2 testing results, and date of death. Data on the following comorbidities were collected: hypertension, type 2 diabetes, ischemic heart disease, atrial fibrillation, dementia, chronic obstructive pulmonary disease (COPD), cancer, heart failure, stroke, obesity and chronic liver disease. Hospital length of stay (days) was calculated subtracting the date of admission from the date of death. This study was carried out in keeping with the principles of the Declaration of Helsinki. On February 27th 2020, the Italian Government authorized the collection and scientific dissemination of data concerning the COVID-19 epidemic by the ISS and other public health institutions [12].

### Statistical analysis

The prevalence (%) of each disease occurring with and without comorbidities was estimated. The expected prevalence of disease pairs was computed as (prevalence of disease A) × (prevalence of disease B) and compared with the observed co-prevalence (Table S1). Logistic regression models were run to analyze the crude and adjusted (age, sex, number of other diseases) association between those pairs of chronic diseases with O/E values of ≥ 1.5 [10].

## Results

**Fig. 1:**
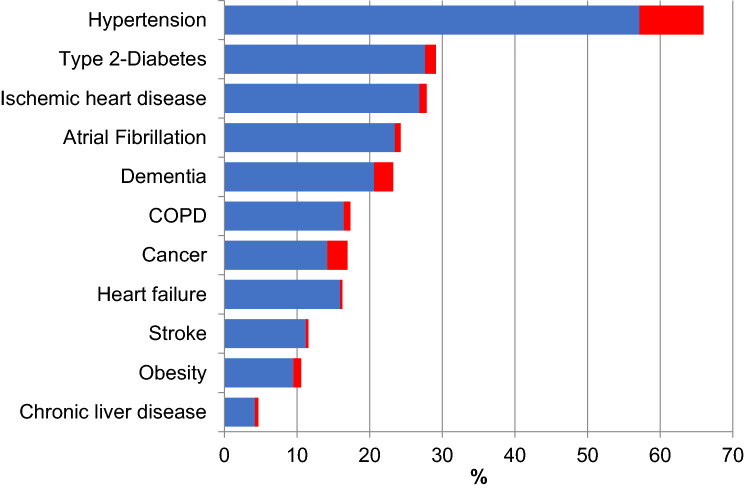
Prevalence per 100 of most frequent chronic diseases co-occurring with other diseases (blue) or without (red). *COPD *chronic obstructive pulmonary disease

The mean age at death of the study participants was 79.1 ± 12.0 years, with the proportion of female participants being 40% and the prevalence of multimorbidity (≥ 2 diseases) reaching as high as 85%. Hypertension (66%), type 2 diabetes (29%), ischemic heart disease (28%), and atrial fibrillation (24%) were the diseases most frequently reported in the clinical records of deceased patients. As depicted in Fig. 1, suffering from one single disease was uncommon; hypertension (8.8%), cancer (2.8%) and dementia (2.7%) were the conditions most frequently reported in isolation. As shown in Table 1, the following disease pairs showed an O/E ≥ 1.5 and statistically significant higher odds of co-occurrence in the crude and adjusted analyses: (1) ischemic heart disease and atrial fibrillation, (2) atrial fibrillation and heart failure, (3) atrial fibrillation and stroke, (4) heart failure and COPD, (5) stroke and dementia, and 6) type 2 diabetes and obesity. In table S2, we reported the median and mean length of stay for each of the disease pairs presenting with an O/E ≥ 1.5. The disease pair diabetes and obesity displayed the shortest length of hospital stay (10.9 days; 95% CI 9.2–12.7), and the pair ischemic heart disease and atrial fibrillation the longest (12.2 days; 95% CI 11.0–13.3).

**Table 1:** Observed and expected frequencies of pairs of chronic diseases in deceased SARS-CoV-2 positive persons, O/E (Observed/Expected) ratios, and crude and adjusted odds ratios for those pairs with O/E > = 1.5

Comorbidities	*N*	Frequency (%)		Ratio O/E	OR (95% CI)	
		Observed	Expected		Crude	Adjusted^a,b^
Ischemic heart disease and atrial fibrillation	570	9.6	6.8	1.8	3.1 (2.7–3.6)	2.6 (2.2–3.0)
Atrial fibrillation and heart failure	472	7.9	4.0	2.0	3.9 (3.4–4.5)	3.1 (2.7–3.6)
Atrial fibrillation and stroke	249	4.2	2.8	1.5	1.9 (1.6–2.3)	1.7 (1.4–2.0)
Heart failure and COPD	258	4.3	2.8	1.5	2.0 (1.7–2.3)	1.6 (1.4–1.9)
Stroke and dementia	248	4.2	2.7	1.6	2.0 (1.7–2.4)	1.8 (1.5–2.2)
Diabetes and obesity	290	4.9	3.1	1.6	2.3 (1.9–2.7)	2.2 (1.8–2.6)

^a^Results from logistic regression models testing the association between pairs of chronic conditions: odds ratios (ORs; crude and adjusted for age, sex, and all the other diseases) and 95% confidence intervals (CI) are reported. *COPD *chronic obstructive pulmonary disease

^b^*p Value* < *0.001* for all adjusted ORs

## Discussion

In this case series of individuals deceased in Italian hospitals with a diagnosis of COVID-19, we found that specific disease combinations including cardiovascular and metabolic conditions as well as dementia occurred more frequently than expected by chance alone. This finding indicates a need to investigate the possible role of these clinical profiles in the chain of events that lead to death in individuals who have contracted SARS-CoV-2.

Globally, the mean age of individuals deceased with COVID-19 has been very high, with nearly all of these persons suffering from coexisting chronic diseases [2, 6]. As shown in several previous population-based studies, multimorbidity can be considered the norm in late life, with a prevalence reaching 90% [13]. While this makes multimorbidity a sensitive tool to predict negative outcomes, its specificity remains debated. For this reason, attention has shifted to the study of specific disease combinations (i.e., clusters) that occur beyond chance and are associated, with higher specificity, to several health-related outcomes. Distinct patterns of multimorbidity have repeatedly been found to be differentially associated with negative health outcomes in the older population [14, 15]. For example, when compared with other disease combinations, multimorbidity clusters including cardiovascular and neuropsychiatric diseases have displayed strong associations with functional impairment, hospitalization, and death [16–18].

Several individual diseases have been identified as clinical substrates of worse COVID-19 prognosis. In particular, heart disease, obesity, cancer, and dementia have been associated with higher odds of hospitalization, intensive care needs, and mortality [19]. The prevalence of such conditions was high in our sample of deceased individuals, supporting the idea that they also play a role in severe COVID-19. In the present study, cardiovascular diseases were involved in five of the six identified disease couples, with atrial fibrillation being part of three of them, and heart failure and stroke included in two of them. The synergy between different cardiovascular diseases appears evident: heart failure, ischemic heart disease, and stroke, coexist and represent common complications of atrial fibrillation. Such a high cardiovascular burden in one individual is arguably responsible for an impaired hemodynamic response to the infection with more severe symptoms triggering the hospitalization, further organ decompensation, and subsequently, higher mortality rate [20]. Another interesting finding is the high likelihood to observe combinations of diseases involving different organs and systems, as heart failure and COPD, and obesity and diabetes. An underlying poor cardiorespiratory fitness in the first case, and the combination of an impaired respiratory mechanics and immunodeficiency in the second case, could be responsible for rapidly evolving COVID-19 cases [21]. Interestingly, the pair diabetes/obesity was associated with the shortest length of hospital stay, so earliest mortality, suggesting a higher lethality of this disease combination among hospitalized patients with COVID-19. Finally, the combination of stroke and dementia emerged as particularly prevalent in our sample population. Dementia often develops as a consequence of stroke, and both have been previously reported as optimal substrates for several infections as well as their complications [22]. This last observation could also reflect the massive burden of COVID-19 on older institutionalized individuals. In our study, it is interesting to note a relative lower prevalence of some disease combinations including obesity—previously reported as a risk factor for COVID-19 mortality and morbidity [21]—as for example the combinations dementia/obesity and stroke/obesity. Arguably, in the context of a selected population of older adults deceased because of COVID-19 the low prevalence of such combinations might be explained by the fact that for both stroke and dementia, an advanced disease is associated with severe nutritional problems, which are unlikely associated with obesity. A somewhat similar paradox emerges observing the O/E values of disease combinations including hypertension, found always to be very close to one, which one more time points at the different clinical significance of specific diseases in the context of old frail individuals, in spite of their high prevalence in absolute terms.

Several limitations should be considered when reading these findings. First, only including individuals who died in hospitals limits the generalizability of the results, making them less applicable to groups of people who died at home or in care homes. Second, if a more extensive list of diseases had been assessed, it is possible that a higher number of meaningful disease combinations could have been uncovered. However, the most prevalent diseases in older adults, and those previously implicated in the infection prognosis, have been considered. Third, with this data it was not possible to compare the comorbidity statuses of deceased and non-deceased COVID-19 in-patients. Finally, the extraction of data derived from clinical charts filled in by different specialists throughout Italy could have introduced some misclassification bias in the diagnosis attribution carried out by the researchers.

In conclusion, disease combinations involving multiple cardio-respiratory, metabolic, and neuropsychiatric diseases occur more frequently than expected in individuals who died due to COVID-19. These same combinations might represent substrates of worse infection and contribute to the chain of clinical events that lead to death. The prompt identification of such individuals could lead to more effective protective strategies such as immunization and social protection.

## Supplementary Information

Below is the link to the electronic supplementary material.

Supplementary file1 (DOCX 24 KB)
