# Supplementary material for: Comorbidity status of deceased COVID-19 in-patients in Italy
Source: Aging Clin Exp Res. 2021 Jun 24;33(8):2361–5. doi: 10.1007/s40520-021-01914-y (PMC8224257; doi:10.1007/s40520-021-01914-y)
Supplement: Supplementary file 1 — Supplementary file1 (DOCX 24 KB) [file 40520_2021_1914_MOESM1_ESM.docx]

**Table S1** - Observed and Expected frequencies of possible pairs of diseases in deceased SARS-CoV-2 positive persons

|  | ***N*** | ***%*** |  |  |
| --- | --- | --- | --- | --- |
|  |  |  |  |  |
| ***Sample size*** | ***6085*** | ***100.0*** |  |  |
| *Missing* | *123* | *2.0* |  |  |
|  |  |  |  |  |
| ***Possible pairs of diseases*** |  |  |  |  |
|  |  | ***Observed, %*** | ***Expected, %*** | ***O/E*** |
|  |  |  |  |  |
| ***Ischemic heart disease*** | ***1661*** | ***27.9*** |  |  |
| *IHD+AF* | *570* | *9.6* | *6.8* | *1.4* |
| *IHD+HF* | *478* | *8.0* | *4.5* | *1.8* |
| *IHD+STROKE* | *238* | *4.0* | *3.2* | *1.2* |
| *IHD+HYPBP* | *1229* | *20.6* | *18.4* | *1.1* |
| *IHD+DIAB* | *605* | *10.1* | *8.1* | *1.2* |
| *IHD+DEM* | *378* | *6.3* | *6.5* | *1.0* |
| *IHD+COPD* | *391* | *6.6* | *4.8* | *1.4* |
| *IHD+CANCER* | *226* | *3.8* | *4.7* | *0.8* |
| *IHD+LIVER* | *77* | *1.3* | *1.3* | *1.0* |
| *IHD+OBESITY* | *160* | *2.7* | *2.9* | *0.9* |
|  |  |  |  |  |
| ***Atrial Fibrillation*** | ***1448*** | ***24.3*** |  |  |
| *AF+HF* | *472* | *7.9* | *4.0* | *2.0* |
| *AF+STROKE* | *249* | *4.2* | *2.8* | *1.5* |
| *AF+HYPBP* | *1065* | *17.9* | *16.0* | *1.1* |
| *AF+DIAB* | *426* | *7.1* | *7.1* | *1.0* |
| *AF+DEM* | *405* | *6.8* | *5.7* | *1.2* |
| *AF+COPD* | *328* | *5.5* | *4.2* | *1.3* |
| *AF+CANCER* | *240* | *4.0* | *4.1* | *1.0* |
| *AF+LIVER* | *67* | *1.1* | *1.1* | *1.0* |
| *AF+OBESITY* | *124* | *2.1* | *2.6* | *0.8* |
|  |  |  |  |  |
| ***Heart Failure*** | ***970*** | ***16.3*** |  |  |
| *HF+STROKE* | *128* | *2.1* | *1.9* | *1.1* |
| *HF+HYPBP* | *726* | *12.2* | *10.7* | *1.1* |
| *HF+DIAB* | *323* | *5.4* | *4.7* | *1.1* |
| *HF+DEM* | *230* | *3.9* | *3.8* | *1.0* |
| *HF+COPD* | *258* | *4.3* | *2.8* | *1.5* |
| *HF+CANCER* | *143* | *2.4* | *2.8* | *0.9* |
| *HF+LIVER* | *57* | *1.0* | *0.8* | *1.3* |
| *HF+OBESITY* | *102* | *1.7* | *1.7* | *1.0* |
|  |  |  |  |  |
|  |  |  |  |  |
|  |  |  |  |  |
|  |  | ***Observed, %*** | ***Expected, %*** | ***O/E*** |
|  |  |  |  |  |
| ***Stroke*** | ***691*** | ***11.6*** |  |  |
| *STROKE+HYPBP* | *510* | *8.6* | *7.6* | *1.1* |
| *STROKE+DIAB* | *218* | *3.7* | *3.4* | *1.1* |
| *STROKE+DEM* | *248* | *4.2* | *2.7* | *1.6* |
| *STROKE+COPD* | *132* | *2.2* | *2.0* | *1.1* |
| *STROKE+CANCER* | *91* | *1.5* | *2.0* | *0.8* |
| *STROKE+LIVER* | *30* | *0.5* | *0.5* | *0.9* |
| *STROKE+OBESITY* | *44* | *0.7* | *1.2* | *0.6* |
|  |  |  |  |  |
| ***Hypertension*** | ***3934*** | ***66.0*** |  |  |
| *HYPBP+DIAB* | *1317* | *22.1* | *19.2* | *1.2* |
| *HYPBP+DEM* | *895* | *15.0* | *15.4* | *1.0* |
| *HYPBP+COPD* | *713* | *12.0* | *11.5* | *1.0* |
| *HYPBP+CANCER* | *615* | *10.3* | *11.2* | *0.9* |
| *HYPBP+LIVER* | *165* | *2.8* | *3.1* | *0.9* |
| *HYPBP+OBESITY* | *466* | *7.8* | *7.0* | *1.1* |
|  |  |  |  |  |
| ***Diabetes*** | ***1739*** | ***29.1*** |  |  |
| *DIAB+DEM* | *318* | *5.3* | *6.8* | *0.8* |
| *DIAB+COPD* | *322* | *5.4* | *5.1* | *1.1* |
| *DIAB+CANCER* | *244* | *4.1* | *4.9* | *0.8* |
| *DIAB+LIVER* | *92* | *1.5* | *1.4* | *1.1* |
| *DIAB+OBESITY* | *290* | *4.9* | *3.1* | *1.6* |
|  |  |  |  |  |
| ***Dementia*** | ***1387*** | ***23.3*** |  |  |
| *DEM+COPD* | *205* | *3.4* | *4.0* | ***0.8*** |
| *DEM+CANCER* | *161* | *2.7* | *3.9* | ***0.7*** |
| *DEM+LIVER* | *64* | *1.1* | *1.1* | ***1.0*** |
| *DEM+OBESITY* | *58* | *1.0* | *2.5* | ***0.4*** |
|  |  |  |  |  |
| ***COPD*** | ***1036*** | ***17.4*** |  |  |
| *COPD+CANCER* | *192* | *3.2* | *2.9* | *1.1* |
| *COPD+LIVER* | *59* | *1.0* | *0.8* | *1.2* |
| *COPD+OBESITY* | *150* | *2.5* | *1.8* | *1.4* |
|  |  |  |  |  |
| ***Cancer*** | ***1011*** | ***17.0*** |  |  |
| *CANCER+LIVER* | *60* | *1.0* | *0.8* | *1.3* |
| *CANCER+OBESITY* | *76* | *1.3* | *1.8* | *0.7* |
|  |  |  |  |  |
| ***Chronic Liver Disease*** | ***280*** | ***4.7*** |  |  |
| *LIVER+OBESITY* | *27* | *0.5* | *0.5* | *1.0* |

**Table S2.** Mean and median time (days) from hospital admission to death of the sample population.

|  |  | |  |  | |
| --- | --- | --- | --- | --- | --- |
| ***Comorbidities*** | **Median** | ***IQR*** |  | **Mean** | ***95% CI*** |
|  |  |  |  |  |  |
| *All* | 7 | *3-14* |  | 11.8 | *11.4-12.2* |
| *Ischemic heart disease and atrial fibrillation* | 8 | *4-16* |  | 12.2 | *11.0-13.3* |
| *Atrial fibrillation and heart failure* | 7 | *3-15* |  | 11.7 | *10.4-12.9* |
| *Atrial fibrillation and stroke* | 8 | *4-15* |  | 11.4 | *9.8-13.0* |
| *Heart failure and COPD* | 7 | *3-14* |  | 11.8 | *10.0-13.5* |
| *Stroke and dementia* | 8 | *4-15* |  | 11.1 | *9.6-12.7* |
| *Diabetes and obesity* | 7 | *3-12* |  | 10.9 | *9.2-12.7* |
